# Transcriptome and proteome depth analysis indicate ABA, MAPK cascade and Ca^2+^ signaling co-regulate cold tolerance in *Rhododendron chrysanthum* Pall.

**DOI:** 10.3389/fpls.2023.1146663

**Published:** 2023-02-21

**Authors:** Qingyi Zhang, Yue Li, Kun Cao, Hongwei Xu, Xiaofu Zhou

**Affiliations:** Key Laboratory of Plant Resource Science and Green Production of Jilin Province, Jilin Normal University, Siping, China

**Keywords:** cold stress, *Rhododendron chrysanthum* Pall., proteome, transcriptome, abscisic acid, MAPK cascade, calcium ion

## Abstract

**Introduction:**

Cold stress is a global common problem that significantly limits plant development and geographical distribution. Plants respond to low temperature stress by evolving interrelated regulatory pathways to respond and adapt to their environment in a timely manner. *Rhodoendron chrysanthum* Pall. (*R. chrysanthum*) is a perennial evergreen dwarf shrub used for adornment and medicine that thrives in the Changbai Mountains at high elevations and subfreezing conditions.

**Methods:**

In this study, a comprehensive investigation of cold tolerance (4°C, 12h) in *R. chrysanthum* leaves under cold using physiological combined with transcriptomic and proteomic approaches.

**Results:**

There were 12,261 differentially expressed genes (DEGs) and 360 differentially expressed proteins (DEPs) in the low temperature (LT) and normal treatment (Control). Integrated transcriptomic and proteomic analyses showed that MAPK cascade, ABA biosynthesis and signaling, plant-pathogen interaction, linoleic acid metabolism and glycerophospholipid metabolism were significantly enriched in response to cold stress of *R. chrysanthum* leaves.

**Discussion:**

We analyzed the involvement of ABA biosynthesis and signaling, MAPK cascade, and Ca^2+^ signaling, that may jointly respond to stomatal closure, chlorophyll degradation, and ROS homeostasis under low temperature stress. These results propose an integrated regulatory network of ABA, MAPK cascade and Ca^2+^ signaling comodulating the cold stress in *R. chrysanthum*, which will provide some insights to elucidate the molecular mechanisms of cold tolerance in plants.

## Introduction

Cold stress is one of the abiotic stresses that severely limit plant growth and development and is a common environmental factor worldwide (Kidokoro et al., 2022). Low temperature stress has been prone to reduce yields and deteriorate the quality of horticultural products resulting in significant losses of horticultural products worldwide Key Laboratory of Plant Resource Science and Green Production of Jilin Province, Jilin Normal University, Siping, China (Theocharis et al., 2012; Eremina et al., 2016). This is a huge challenge for horticulture and agriculture. Therefore, understanding the molecular modes that support resistance to cold stress is essential to further optimize horticultural and agriculture crop breeding and production.

The plant response to low temperature stress reflects a complex regulatory network for inducing changes in the expression of cold-responsive genes and associated proteins (Wang et al., 2021; Zhang, H. et al., 2022). It has been shown that low temperatures cause changes in plant membrane fluidity and that increased Ca^2+^ levels induce phosphorylation of calcium-dependent protein kinase (CPKs) and mitogen-activated protein kinases (MAPK) cascade-related proteins in response to cold stress (Morrison, 2012). The MAPK cascade consists of three components, MAPK kinase kinases (MAPKKKs)-MAPK kinases (MKKs)-MAPKs, and is highly conserved in eukaryotes (Sinha et al., 2011). Low temperature induces an increase in Ca^2+^ and the signal is transmitted to the Ca^2+^ sensors calcieurin B-like protein (CBL), Calcium-dependent protein kinase (CPKs), calmodulin (CaM) and Ca^2+^/CaM-dependent protein kinase (CCaMK) (Zhu, 2016).

Numerous research conducted in recent years have demonstrated that plant hormones regulate the signaling of the response to cold stress (Zhang, H. et al., 2022). Abscisic acid (ABA) is essential in the induction of stomatal closure, seed dormancy and plant response to abiotic stress (Dong et al., 2015). Increased ABA biosynthesis in rice and Arabidopsis correlated with increased ABA levels under cold stress (Baron et al., 2012; Maruyama et al., 2014). Exogenous application of ABA improved the cold tolerance of the plants (Yu et al., 2020). The Pyrabactin resistance 1(PYR)/PYR1-like (PYL) proteins (PYR/PYL) are recognized as ABA receptors, and binding of PYLs to ABA prevents PP2C from inhibiting SnRK2 and activating complex signaling pathways downstream of ABA (Antoni et al., 2012; Li et al., 2018; Chen et al., 2020). There may be an interaction between SnRK2 kinase and Ca^2+^ signaling involved in ABA signaling to induce stomatal closure (Daszkowska-Golec and Szarejko, 2013; Garcia-Leon et al., 2019).

*Rhododendron chrysanthum* Pall. (*R. chrysanthum*) is a horticultural plant that grows in alpine grassland zones or on moss layers at altitudes of 1,000-2,506 m and has some medicinal value (Zhou et al., 2021; Sun et al., 2022). Our previous studies have provided physiological evidence for the plant strong ability to withstand low temperature stress (Zhou et al., 2017). For the first time, the Illumina HiSeq platform was used to sequence the *R. chrysanthum* transcriptome under cold stress and construct cDNA libraries to identify plant hormone and signal transduction pathways, TFs, lipid metabolism and Ca^2+^ signaling genes in response to cold stress (Cao et al., 2022; Zhang, Q. et al., 2022). By TMT/iTRAQ quantitative phosphoproteome, we found that 274 significantly changed proteins indicated changed signal transduction and energy conversion, Ca^2+^ signaling, TFs and reactive oxygen species (ROS) homeostasis could protect *R. chrysanthum* from prevents damage caused by low temperatures (Liu et al., 2022). As a result, the *R. chrysanthum* is a crucial plant resource for the investigation of plant tolerance. However, combined transcriptomic and proteomic insights are essential for a comprehensive understanding of the defense mechanisms of low temperature stress in *R. chrysanthum*.

In the present study, we used cold-treated and control *R. chrysanthum* as material to explore the molecular mechanisms at the transcriptome and proteome levels and to search for key genes and proteins in response to cold stress. Therefore, we analyze global transcripts and proteins under cold stress. We suggest a regulatory network made up of the MAPK cascade, Ca^2+^ signaling, and the ABA signaling pathway to investigate the mechanism of *R. chrysanthum*’s cold tolerance.

## Materials and methods

### Plant material and cold treatments

We collected *R. chrysanthum* on Changbai Mountain at altitudes between 1,300 and 2,650 m. We selected 8 months old seedlings of essentially uniform growth as the test material (Zhang, Q. et al., 2022). The plants were grown in an artificial climate room at 25° (14-h light)/25 ° (10-h dark) under a 50 µmol (photon) m^−2^s^−1^ white fluorescent light. After low temperature treatment at 4° 12 h (LT) application, which in the artificial climate room 12 h as the control sample (Control). The experiment was repeated three times. Leaf samples are collected and immediately frozen in liquid nitrogen for subsequent sequencing.

### Physiological measurements of *R. chrysanthum*

200 mg of leaves from each *R. chrysanthum* were taken for the determination of enzymatic activity, and six samples were processed according to the method of the corresponding kit. We assayed SOD (EC 1.15.1.1) and CAT (EC 1.11.1.6) activities in the leaves according to the previous method (Zhao et al., 2016). Leaf photosynthetic parameters were determined using the CIRAS-3 portable photosynthesis measurement system (PP Systems, Inc., USA). Stomatal traits were determined by reference to the method of Montillet (Montillet et al., 2013). Protocells were made and observed under a light microscope 40x (Nikon ECLIPSE Ti-S). The expression level method was used to calculate each transcript’s gene expression level in order to compare the levels of protein and gene expression between LT and Control. Bradford analysis was used for protein quantification. After matching clean reads to genomic sequences using Bowtie2, we used RSEM to calculate the gene expression levels for each sample.

### cDNA library construction and transcriptomics data analysis

The mRNA enrichment approach was used to process total RNA (Li and Dewey, 2011). Utilizing Oligo(dT) magnetic beads and the Oligitex mRNA kit, PolyA tailed mRNA was isolated. First-strand cDNA was produced by adding the proper quantity of lysis agent at a high temperature and using the cleaved mRNA as a template. The synthesis reaction system was then set up to produce second-strand cDNA, which was then purified, recovered, and repaired using the kit. The mucus end of the purified cDNA is joined to the “a” at the “3” end. The final PCR amplification and the size of the fragment after modification are used to determine the product. Library quality was assessed using an Agilent 2100 Bioanalyzer and an ABI StepOnePlus RealTime PCR system. Transcriptome sequencing for this investigation was carried out using the IlluminaHiSeq Platform, which is based on Shenzhen UW Genetic Technology Research Co. To ensure the quality and accuracy of the data, SOAPnuke was used to filter and remove contamination, low quality reads containing position bases greater than 5% and bases with quality values lower than 10 as a percentage of the total bases in that reads greater than 20%. The clear reads were compared to the reference gene sequences using Bowtie2, and clear reads were assembled using Trinity reads were assembled *de novo* using Trinity to obtain unigene.

### Protein extraction and trypsin digestion

Liquid nitrogen was used to grind the leaves into a cell powder, which was then transferred to a 5 mL centrifuge tube. Four volumes of lysis buffer (8 M urea, 1% Triton-100, 10 mM dithiothreitol, and 1% protease inhibitor cocktail) were added to the ground cell material and sonicated three times on ice using a high intensity sonication processor (Scientz). Centrifugate the leftover waste at 20,000 g for 10 min. at 4°C. The protein should next be precipitated with cold 20% TCA at -20°C for 2 hours. Following centrifugation at 12,000 g for 10 min at 4°C, the supernatant was discarded. Three cold acetone washes were applied to the residual precipitate. The proteins were redissolved in 8 M urea as per the manufacturer’s instructions, and the protein concentration was determined using the BCA kit.

### TMT/iTRAQ labeling and LC-MS/MS analysis

Peptides are desalted by a Strata X C18 SPE column after trypsin digestion and then dried under vacuum. The peptide mixture was mixed, desalted and dried by vacuum centrifugation after incubation for two hours at room temperature with one unit of TMT/iTRAQ reagent, which had been thawed and reconfigured in acetonitrile. The soluble proteome of LT and control and their respective three biological replicates were analyzed using LC-MS/MS. Samples were dissolved in 0.1% formic acid and loaded onto a reversed-phase analytical column for analysis. The methods used for sample preparation and equipment setup were similar to those mentioned previously (Xu et al., 2021).Tandem mass spectrometry (MS/MS) was performed by on-line connection to a UPLC with Q ExactiveTM Plus (Thermo Fisher Scientific, USA) at an applied electrospray voltage of 2.0 kV. The mass spectrometer and orbiter were used to find intact peptides. The final MS/MS data were processed with the Maxquant (v.1.5.2.8) search engine.

### Identification and analysis of differentially expressed genes (DEGs) and proteomes

The DEseq R package was used in tests to find DEGs in *R. chrysanthum* in response to cold stress. In this study, genes with the false discovery rate (FDR) < 0.05 and |log (fold change) | ≥ 1 were DEGs, and proteins with fold change > 1.3 and FDR-corrected *p* value of 0.05 were defined as differentially expressed proteins.

### Database search and statistical analysis

Protein pathways were annotated using the KEGG (Kyoto Encyclopedia of Genes and Genomes) database. The UniProt-GOA database served as source for the Gene Ontology (GO) annotation proteome. R software (http://cran.R-project.org/) and IBM SPSS 22.0 (NY, USA) software were both used to conduct the statistical analyses.

## Results

### Physiological and biochemical responses under cold stress

**Figure 1:**
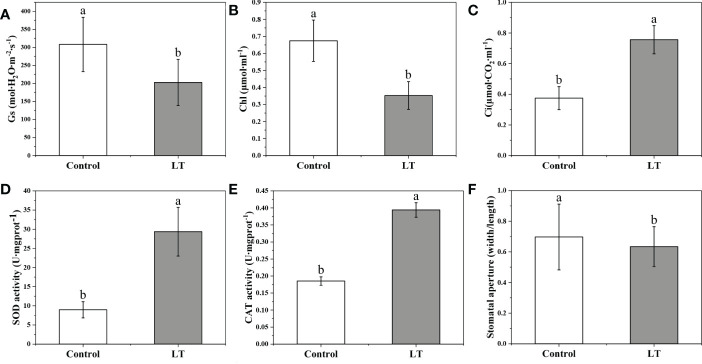
Physiological and biochemical reaction to the low temperature stress in *Rhododendron chrysanthum* Pall. **(A)** Stomatal conductance (Gs). **(B)** Chlorophyll contents (Chl). **(C)** Intercellular CO_2_ concentration (Ci). **(D)** Superoxide dismutase (SOD) activity activities. **(E)** Activity of the enzyme catalase (CAT). **(F)** Stomatal aperture (width/length). Control, samples that culture under 25°C; LT, freezing treatment samples under 4°C 12 h; Means ± SD are used to express values. The varied letters among the various treatments represent statistically different values (*p*<0.05).

To evaluate the cold tolerance in *Rhododendron chrysanthum* Pall. (*R. chrysanthum*), we measured physiological and biochemical responses in control group and low temperature(LT) samples of *R. chrysanthum* leaves. Stomatal conductance (Gs) also decreased in response to the cold stress, a 34.22% decrease in Gs compared to Control (**Figure 1**). The results showed a significant decrease of 50.57% in LT chlorophyll content compared to the control group (**Figure 1**). Besides these, Ci increased significantly and stomatal aperture was significantly narrower. SOD is a class of enzymes that catalyze the conversion of superoxide anion radical to H_2_O_2_ and O_2_. CAT can effectively prevent the over-accumulation of ROS and remove the H_2_O_2_. The analysis of antioxidant enzyme activity revealed that SOD, CAT were responded to low temperature stress (**Figure 1E**). Compared to the Control, the SOD and CAT activities of LT increased by 3.27- and 2.13-fold, respectively.

### Transcriptomics analysis of *R. chrysanthum* leaves in response to cold stress

**Figure 2:**
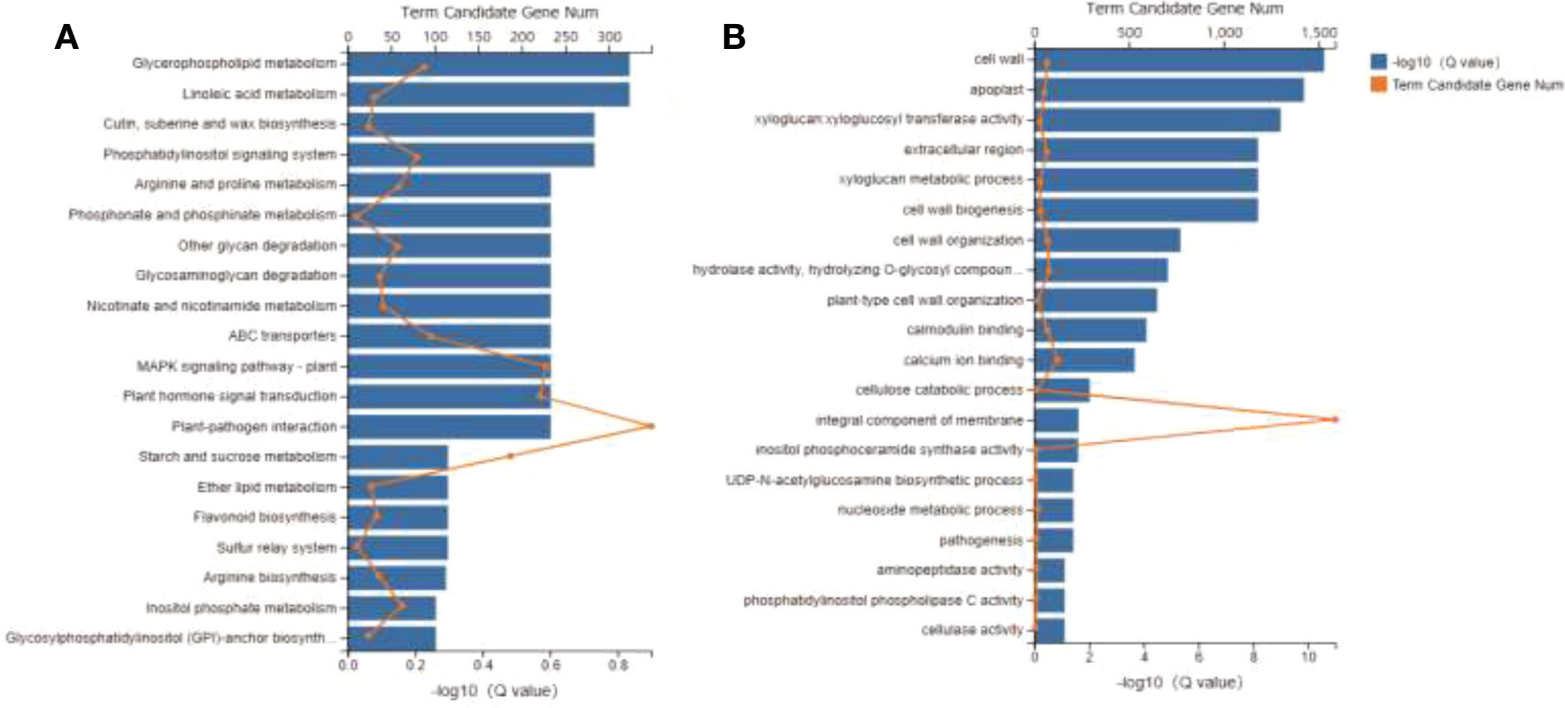
Global transcriptome of *Rhododendron chrysanthum* Pall. in response to cold stress. **(A)** KEGG classification of all DEGs annotated. **(B)** DEG enrichment through GO.

Transcriptomics analysis was utilized to detect the DEGs in the leaves treated with LT and Control in order to understand the molecular basis of the cold tolerance in *R. chrysanthum*. A total of 254.88 million clean reads were generated for this investigation, along with 66,222 transcripts and 56,145 functionally identified unigenes. The DEGs were discovered using the DESeq2 software, | log2 (fold change) |≥ 1 and p<0.05, and a pairwise comparison between the LT and Control group. Among them, 6,811 decreased and 5,450 upregulation DEGs under cold stress (**Supplementary Figure 1**). These genes were mostly classified by KEGG as being involved in starch and sucrose metabolism, plant hormone signal transduction, plant MAPK signaling pathway, and plant-pathogen interaction (**Figure 2A**). The GO enriched mainly in integral component of membrane, calcium ion binding, cell wall organization and cell wall (**Figure 2B**). This suggests that these pathways may constitute potential molecular mechanisms involved in the response of *R. chrysanthum* to low temperature stress.

### Cold stress responsive proteome in *R. chrysanthum* leaves

**Figure 3:**
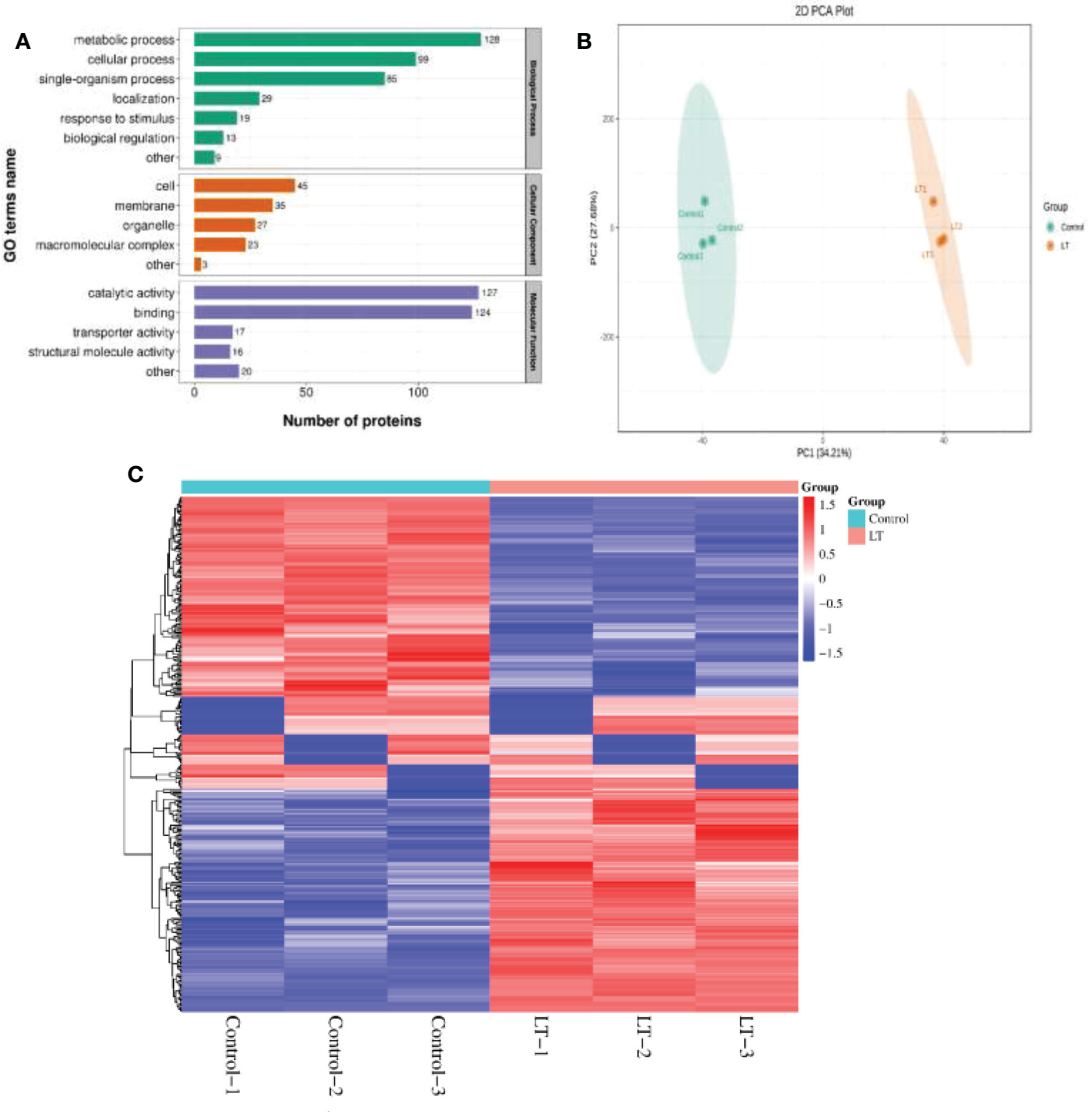
Global proteome in response to cold stress of *R. chrysanthum*. **(A)** GO enrichment of total DEPs. Protein numbers are displayed above each bar. **(B)** The divergence of the corresponding proteomes in response to LT and Control is shown by PCA. **(C)** In comparison to Control samples, DEP abundance under low temperature stress was used to create hierarchically clustered groups.

TMT/iTRAQ quantitative proteome analysis was used to examine the cold stress sensitive protein in *R. chrysanthum* leaves. In total, 6,378 protein species in leaves were identified in these independent biological replicates, 360 of which were defined as DEPs. Furthermore, the DEPs shared 175 protein species increased and 185 decreased under low temperature stress (**Supplementary Figure S1B**). The DEPs were GO enrichment classified into biological process, cellular component and molecular function (**Figure 3**). The DEPs demonstrated that these detections highlighted the enrichment in metabolic process (128), cellular process (99) and single-organism process (85) into biological process; cell (45), membrane (35) and organelle (27) into cellular component; catalytic activity (127) and binding (124) into molecular function. The PCA plot demonstrated that PC1 (34.21%) clearly distinguished the LT group from the Control group, and PC2 explained 27.68% (**Figure 3**). We clustered DEPs hierarchically according to their expression abundance in the LT and Control groups. Hierarchical clustering analysis showed that the DEPs samples from the proteome of the LT group were enriched, which classified into the same clusters (**Figure 3**).

### Integrated analysis of abscisic acid in *R. chrysanthum* leaves under cold stress

**Figure 4:**
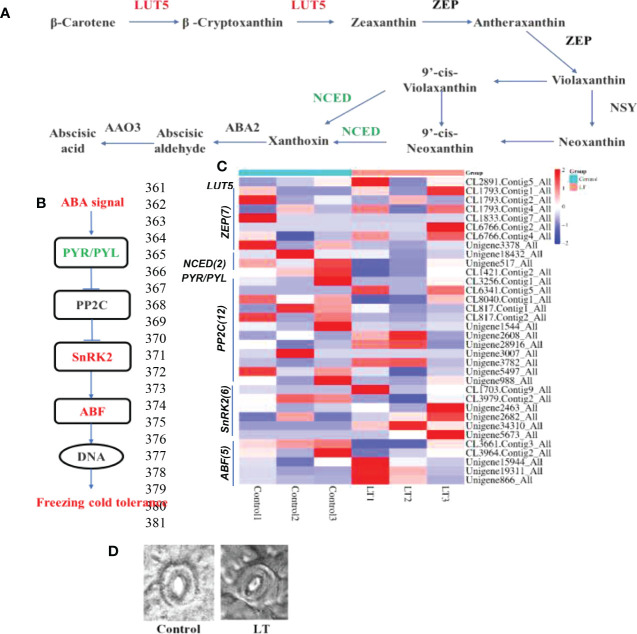
The response of ABA biosynthesis and signaling pathway to cold stress. **(A)** The KEGG was used to build the ABA biosynthetic pathway. **(B)** The pathway of ABA signaling transduction. **(C)** Heat map clustering analysis of ABA biosynthesis and ABA signaling genes after cold stress according to their expression levels. **(D)** In the control and LT groups, the abaxial epidermal stomatal morphology of *R. chrysanthum* leaves.

The *LUT5* and most of *ZEP* showed a significantly increased expression in LT group. Interestingly, the *NCED* was downregulation under cold stress, which is an essential enzyme involved in ABA catabolism (**Figure 4C**). This may contribute to the accumulation of ABA in *R. chrysanthum* leaves after cold stress. We conducted an integrated analysis of changes in ABA production and signaling pathway gene expression of *R. chrysanthum* leaves under cold stress since ABA plays a crucial role in the response to abiotic stress in plants. We found that carotenoid biosynthesis was induced after low temperature stress. This pathway is involved in the biosynthesis of ABA. In this study, 24 ABA receptors were differentially expressed in LT group. The *PYR/PYL* was downregulated. And the 12 *PP2C* genes were detected, most were downregulated by chilling. Five of the six SnRK2 genes were significantly up-regulated under cold stress. Five *ABF* genes were identified, and most were displayed increased in LT group (**Figure 4**).

Increased ABA levels in plants under stress conditions can be involved in the induction of leaf stomatal closure. Furthermore, we assessed the stomatal length-width ratio and investigated at stomatal closure (**Figure 4**). The stomatal aperture was significantly reduced under low temperature stress (**Figure 1**). This may be related to the accumulation of ABA under cold treatment to induce stomatal closure and improve the cold tolerance of *R. chrysanthum*.

### The MAPK signaling pathway to freezing cold stress in *R. chrysanthum* leaves

**Figure 5:**
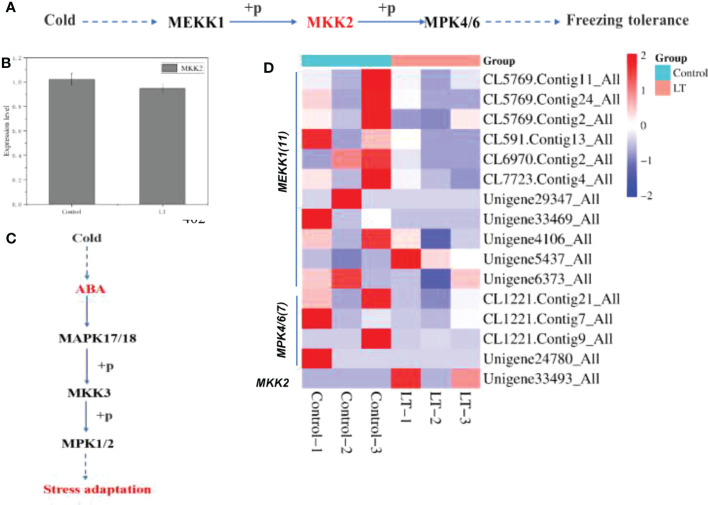
Sensing cold signal related metabolic pathway of *R. chrysanthum*. **(A)** MAPK signaling pathway to freezing cold stress. **(B)** The expression of MKK2 was down-regulated under cold stress. **(C)** ABA regulates the adaptation of plant MAPK signaling pathway to stress under cold stress. **(D)** Heat map clustering analysis of MAPK signaling pathway genes after cold stress according to their expression levels.

In this study, the MAPK signaling pathway was enriched to 19 DEGs (**Figure 5D**). Of these, cold stress resulted in a significant up-regulated of 3 DEGs and 15 DEGs were significantly down-regulated in the LT group (**Figure 5**). Most DEGs involved in pathway lead to response to low temperature stress. The pathway genes *MEKK1* (1 transcripts), *MKK2* (1 transcripts), *MAPK4/6* (1 transcripts) showed significantly increased expression when the samples were exposed to cold stress (**Figure 5D**). And the most DEGs and DEPs were significantly decrease. Notably, quantitative analysis of relevant proteins using TMT/iTRAQ showed a decrease in MKK2 expression in the LT group compared to the control group (**Figure 5**).

### Calcium signaling pathway combined with MAPK cascade and nitric oxide signaling pathway in response to cold stress of *R. chrysanthum* leaves

**Figure 6:**
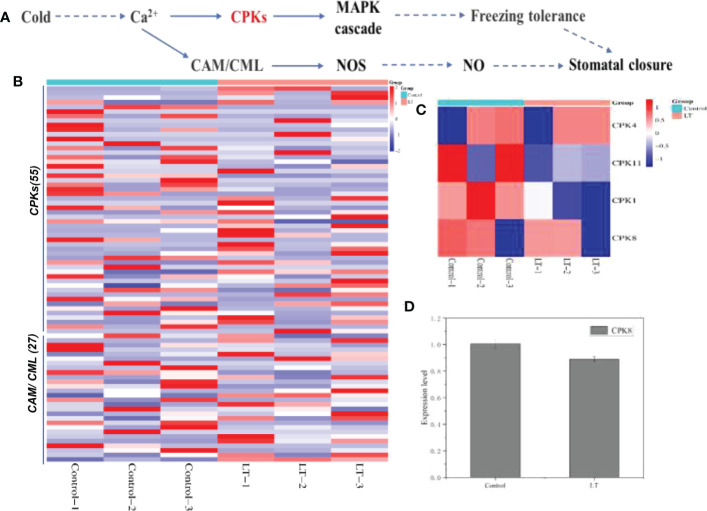
Response of stomatal closure induced by calcium ion signal to freezing stress. **(A)** The KEGG database was used to construct the plant-pathogen interaction. **(B)** Based on the level of expression level, the DEGs of calcium signal transduction under cold stress were hierarchically clustered. **(C)** On the basis of their expression level, the DEPs of CPKs were hierarchically clustered. **(D)** The expression of CPK8, a key protein induced stomatal closure, was down-regulated under cold stress.

In the comprehensive analysis, we found that the calcium signaling pathway (ko09130) combined with MAPK cascade and nitric oxide signaling pathway were modulated by low temperature stress. What attracted attention was that Ca^2+^ as second messengers activating downstream related gene expression following cold stress (**Figure 6**). Ca^2+^ transmits cold signals through the interaction of CPKs, CAMs and we screened *CPKs* (55 transcripts), *CAM/CML* (27 transcripts) for DEGs in the LT and control groups (**Figure 6B**). Two of the genes encoding CPK3 and two encoding CPK8 were significantly up-regulated and one encoding CPK4 was significantly down-regulated (**Figure 6**). In proteomic analysis, CPK1/4/8/11 expression was decreased after cold stress (**Figure 6**). Of interest, CPK8 expression was significantly reduced by 1.13-fold after cold stress (**Figure 6**).

## Discussion

### Calcium-mediated signaling pathways in response to cold stress

Low temperature stress can precisely induce physical or chemical changes in plant intracellular biomolecules that trigger stress responses (Sangwan et al., 2002). Plant cells adapt to temperature changes through specific molecular cellular pathways, which include Ca^2+^-mediated signaling. Modulators of Ca^2+^ channels or Ca^2+^ channels located in the plasma membrane are currently several possible cold stress receptors whose functional defects result in the inability of plants to sense cold (Ma et al., 2015; Liu et al., 2021). For example, COLD1 in the rice plasma membrane and endoplasmic reticulum binds to G protein α subunit 1 and is capable of mediating cold signaling (Ma et al., 2015). We discovered that numerous CPKs and CAM/CML genes significantly increased following exposure to cold (**Figure 6B**). CBL1 and CIPK7 are implicated in the response to cold stress, and CBL and CBL-interacting protein kinases (CIPK) form a complex (Huang et al., 2011). In our study, eight CPK28 genes were found to be significantly up-regulated in the LT group (**Supplementary Table 1**), which may be involved in the regulatory network of *R. chrysanthum* leaves in cold tolerance. The *Arabidopsis* CPKs family contains 34 genes that are involved in how plants respond to abiotic stress (Hrabak et al., 2003; Asano et al., 2012). And in rice, 29 CPK genes have been discovered (Asano et al., 2005). *CPK4* (1 transcripts) was identified as down-regulated in *R. chrysanthum* leaves under cold stress, along with a significant decrease in CPK4 content in the proteome (**Figure 6C**). Related reports demonstrate the role of CPK8 in ABA-mediated stomatal closure in response to drought stress (Zou et al., 2015). Interestingly, most *CPK8* (3 transcripts) in *R. chrysanthum* leaves was significantly decreased under low temperature stress and CPK8 protein expression was significantly down-regulated (**Figure 6D**). Heat tolerance of *cpk28* is significantly reduced and phosphorylation of ascorbate peroxidase 2 (APX2) by CPK28 improves heat tolerance in tomato(Hu et al., 2021). CPK28 initiates a phosphorylation cascade that nuclear translocation of nin-like protein 7 (NLP7) and induces transcriptional reprogramming downstream of Ca^2+^ signaling of *Arabidopsis* in response to cold stress (Ding et al., 2022).

### MAPK cascade is involved in ABA signaling in response to cold stress

In our study, 1.15-fold increase in MPK3 after cold stress, but 1.08-fold decrease in MKK2 (**Figure 5C**). Therefore, we speculate that MPK3 participated of *R. chrysanthum* leaves respond to cold stress. Most of the SnRK2 and ABF were significantly upregulated in LT group, which may be related to the MAPK cascade being activated. One of the primary channels for receiving signals from outside cellular stimuli, the MAPK cascade is crucial for plant resistance to abiotic stress (Lee et al., 2016). After environmental changes, MAPK cascade can directly or indirectly participate in ABA signaling, and induce stomatal closure to reduce stress damage to plants (Jammes et al., 2009; Montillet et al., 2013). ABA content increased under cold stress, ABA signaling by PYR/PYL and activated SnRK2 to induce stomatal closure (Hewage et al., 2020). However, SnRK2.6/OST1 was able to be activated of ABA-independent pathway under cold stress (Ding et al., 2015). ABA controls a substantially higher percentage of protein-coding genes in Arabidopsis than do other hormones—nearly 10% of them (Fujita et al., 2006; Cutler et al., 2010). It has been demonstrated that ROS can activate the MAPK cascade, suggesting that ABA and ROS signaling may work in concert to cause stomatal closure at the MAPK level (Zhang et al., 2007). In addition, when MAPKK inhibitor was given to pea epidermis, ABA-induced stomatal closure was prevented (Burnett et al., 2000). MPK4/9/12 and MKK2 were found in guard cells of *Arabidopsis*, and MPK3/5/7/18/20 were able to upregulate expression in response to ABA (Zhao et al., 2008).

### Abscisic acid, MAPK cascade and Ca^2+^ signaling may comodulate cold tolerance in *R. chrysanthum* leaves

Temperature is essential for plant growth. Many studies have elucidated the mechanisms of response to cold stress, which can directly inhibit the reprogramming of key metabolic enzymes and gene expression (Ding et al., 2019; Chang et al., 2020). ABA, MAPK cascade, and Ca^2+^ signaling may jointly regulate cold tolerance in *R. chrysanthum* leaves. It was shown that CPK3 and CPK6 have positive regulatory effects on abiotic stress in plants, and are involved in stomatal closure regulated by ABA and guard cell ion channels (Mori et al., 2006). CPK3 may respond to stress signals independently of the MAPK cascade (Mehlmer et al., 2010). In our study, we found that *CPK3* (2 transcripts) and stomatin-like protein 2 (1 transcripts) expression were significantly increased in the LT group (**Supplementary Table 1**). In *Arabidopsis* CPKs phosphorylate ABFs involved in ABA signaling, and CPK4/11 phosphorylate ABF1 and ABF4 are regulating ABA signaling in stomatal movement, seeding growth and tolerance to abiotic stress (Zhu et al., 2007). Abiotic stress and ABA signaling in plants are both positively regulated by CCaMK, which is crucial (Ni et al., 2019). In rice, ABA stimulates the synthesis of H_2_O_2_, which activates CCaMK and causes DMI3 to become active, resulting in the ABA response (Ni et al., 2019). Through the calcium/calmodulin-regulated receptor-like kinase CRLK1, the MEKK1-MKK2-MPK4 cascade in Arabidopsis is connected to cold-induced Ca^2+^ signaling (Yang et al., 2010; Zhao et al., 2017). H_2_O_2_ produced by stress can activate Ca^2+^ signal and phosphorylate SLAC1, which is the effector of ABA response (Brandt et al., 2015). In addition, the MAPK cascade activates K^+^ channel to release K^+^ in response to stress, which is essential for ABA-induced stomatal closure (Isner et al., 2018). Nitric oxide (NO) plays a significant role in controlling plant development and activating cold tolerance mechanisms, and CAM can activate NOS-induced NO synthesis (Kondo et al., 1999; Costa-Broseta et al., 2019; Sun et al., 2021). NO inhibits the degradation of CHL and negatively regulates Chl metabolizing enzyme activity (Bhattacharya et al., 2017). NO inhibits SnRK2.6/OST1 in guard cells, which negatively affects ABA signaling (Wang et al., 2015).

**Figure 7:**
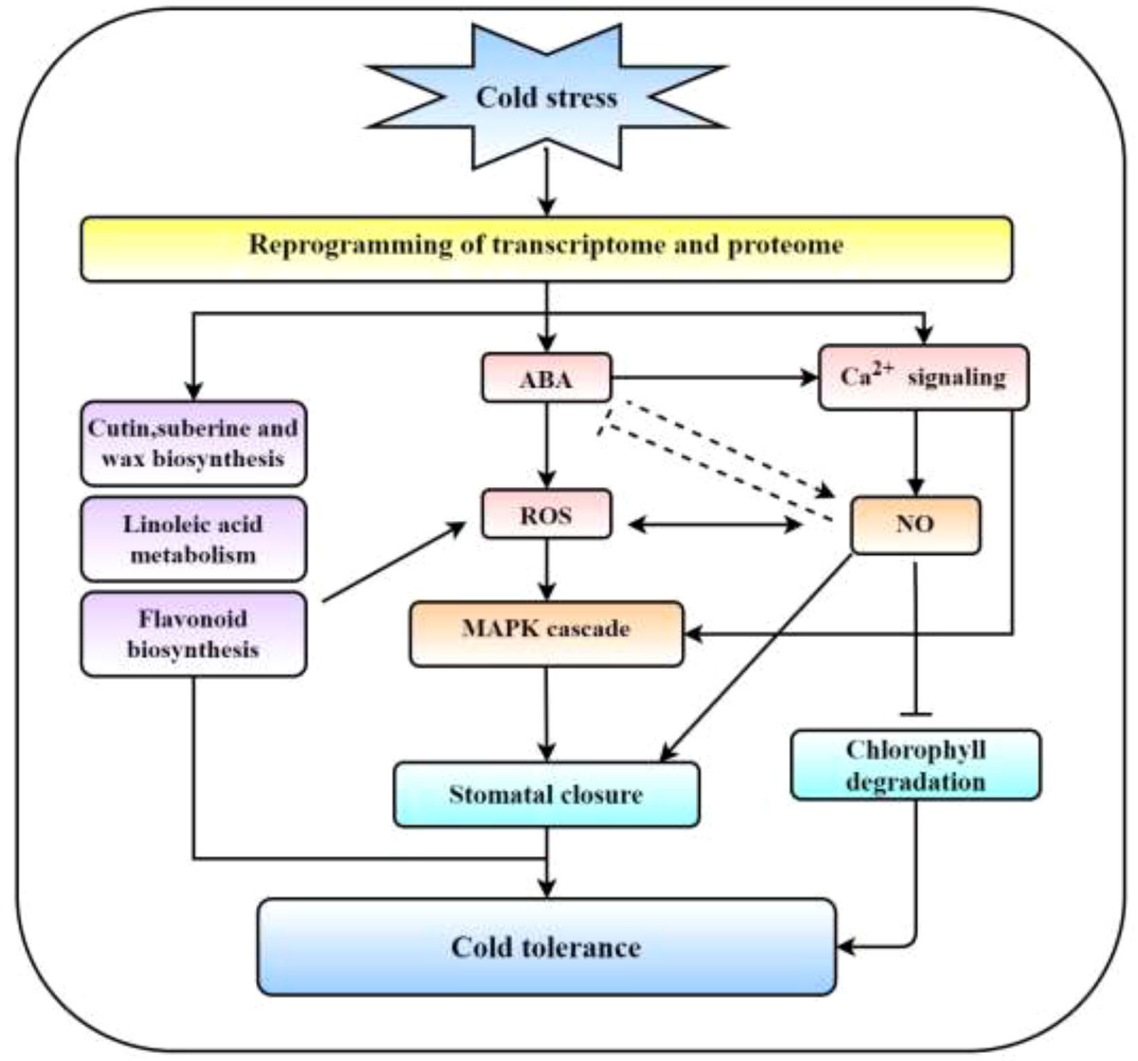
Ca^2+^ signal, ABA, and MAPK cascade comodulated cold tolerance schematic model. Under cold stress, the transcriptome and proteome are reprogrammed, causing significant responses in MAPK cascade, Ca^2+^ signaling, ABA signaling, cutin, suberine and wax biosynthesis, linoleic acid metabolism, NO signaling and flavonoid biosynthesis. Ca^2+^ activates NOS to promote NO production and inhibit Chl degradation. Ca^2+^ signal, ABA and MAPK cascade form the regulatory network and synergistic role in ROS and induction of stomatal closure. These pathways together form a network that regulates cold resistance in *R. chrysanthum*.

In conclusion, we suggest a paradigm in which the cold tolerance of *R. chrysanthum* leaves is comodulated by the ABA, MAPK cascade, and Ca^2+^ signaling (**Figure 7**). Low temperature stress induces transcriptome and proteome reprogramming in *R. chrysanthum* leaves, following the main compelling response pathways of ABA biosynthesis and signal transduction, MAPK signaling pathway, linoleic acid metabolism and flavonoid biosynthesis. The cell membrane senses cold stress and stimulate Ca^2+^ signaling. CAM activates NOS to induce NO production and regulates gene expression and protein activity in response to cold stress. Importantly, these pathways together form a network that regulates cold resistance in *R. chrysanthum*.

## Conclusion

In this investigation, the putative pathways behind *R. chrysanthum*’s cold tolerance were investigated using a mix of proteome and transcriptome research. In *R. chrysanthum* leaves, 360 DEPs and 12,261 DEGs were found. The MAPK cascade, Ca^2+^ signaling, ABA signaling, cutin, suberine, and wax biosynthesis, linoleic acid metabolism, NO signaling, and flavonoid biosynthesis all significantly change in response to cold stress, which reprograms the transcriptome and proteome. Ca^2+^ activates NOS, promoting NO synthesis and preventing Chl degradation. ROS and the induction of stomatal closure are caused by the regulatory network that is composed of the Ca^2+^ signal, ABA, and MAPK cascade. These pathways together form a network that regulates cold tolerance in *R. chrysanthum*.

## Data availability statement

The original contributions presented in the study are publicly available. This data can be found here: https://www.ncbi.nlm.nih.gov/bioproject/PRJNA756577/.

## Author contributions

QZ, YL, KC, HX, and XZ contributed to the experimental design, data recording, data analysis and the preparation and writing of this paper. All authors contributed to the article and approved the submitted version.
