# Supplementary material for: Transcriptome and proteome depth analysis indicate ABA, MAPK cascade and Ca2+ signaling co-regulate cold tolerance in Rhododendron chrysanthum Pall
Source: Front Plant Sci. 2023 Feb 21;14:1146663. doi: 10.3389/fpls.2023.1146663 (PMC9989302; doi:10.3389/fpls.2023.1146663)
Supplement: Supplementary file 1 [file DataSheet_1.docx]

Supplementary Material

# Supplementary Figures and Tables

##
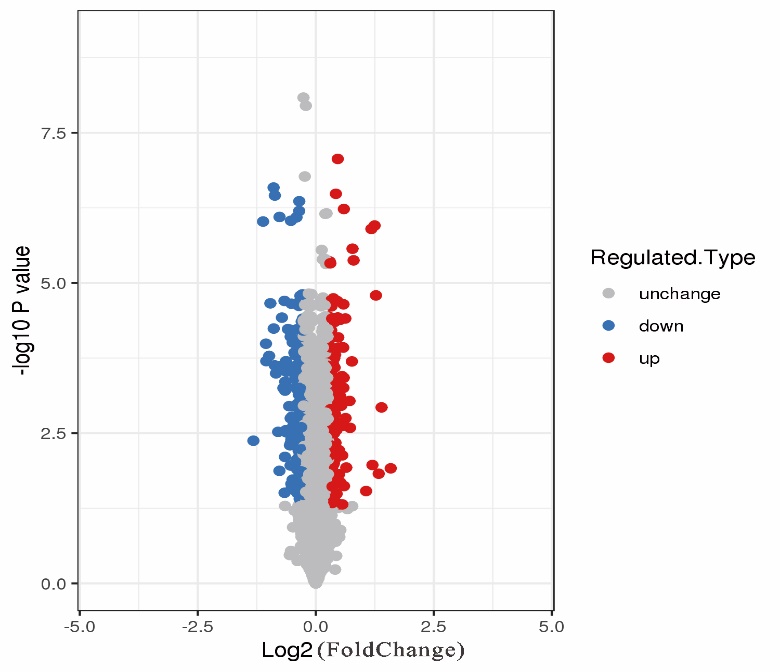

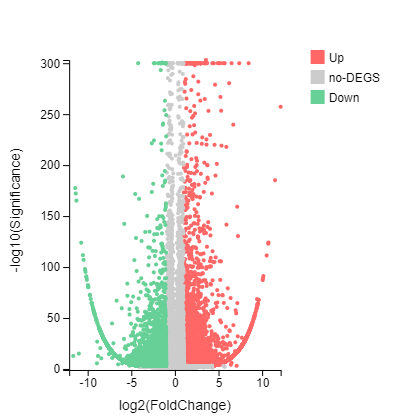
Supplementary Figures

B

A

**Supplementary Figure 1.** **Volcano map of (A) DEGs and (B) DEPs between the LT and the Control group.**

## Supplementary Tables

**Supplementary Table S1. Differentially expressed genes associated with Ca^2+^ signaling proteins in *R. chrysanthum* under cold stress**

| Gene ID | Description | Log_2_(LT/Control) | Gene ID | Description | Log_2_(LT/Control) |
| --- | --- | --- | --- | --- | --- |
| CL1214.Contig1_All | calcium-dependent protein kinase | 4.48 | CL31.Contig17_All | calcium-dependent protein kinase 14 | 1.26 |
| CL31.Contig11_All | calcium-dependent protein kinase 32 | 6.60 | CL31.Contig18_All | calcium-dependent protein kinase 32-like | 1.26 |
| CL31.Contig19_All | calcium-dependent protein kinase 14 | -8.33 | CL456.Contig19_All | calcium-dependent protein kinase 28 | -9.19 |
| CL31.Contig2_All | calcium-dependent protein kinase 32 | -7.76 | CL456.Contig20_All | calcium-dependent protein kinase 28 | 4.42 |
| CL4516.Contig1_All | calcium-dependent protein kinase 2 | -4.00 | CL456.Contig21_All | calcium-dependent protein kinase 28 | -1.99 |
| CL456.Contig11_All | calcium-dependent protein kinase 28 | 7.44 | CL456.Contig23_All | calcium-dependent protein kinase 28 | -2.05 |
| CL456.Contig14_All | calcium-dependent protein kinase 28 | -2.27 | CL456.Contig25_All | calcium-dependent protein kinase 28 | 6.72 |
| CL456.Contig26_All | calcium-dependent protein kinase 28 | -2.08 | CL456.Contig34_All | calcium-dependent protein kinase 28 | -6.77 |
| CL456.Contig28_All | calcium-dependent protein kinase 28 | -1.60 | CL456.Contig35_All | calcium-dependent protein kinase 28 | -3.20 |
| CL456.Contig29_All | calcium-dependent protein kinase 28 | -1.13 | CL456.Contig38_All | calcium-dependent protein kinase 28 | -2.88 |
| CL456.Contig32_All | calcium-dependent protein kinase 28 | 7.97 | CL456.Contig40_All | calcium-dependent protein kinase 28 | -2.98 |
| CL456.Contig33_All | calcium-dependent protein kinase 28 | -4.87 | CL456.Contig41_All | calcium-dependent protein kinase 28 | 5.24 |
| CL456.Contig44_All | calcium-dependent protein kinase 28 | 5.15 | CL456.Contig9_All | calcium-dependent protein kinase 28 | 4.18 |
| CL456.Contig4_All | calcium-dependent protein kinase 28 | -1.91 | CL486.Contig10_All | calcium-dependent protein kinase | -1.20 |
| CL456.Contig6_All | calcium-dependent protein kinase 28 | -2.73 | CL486.Contig20_All | calcium-dependent protein kinase | 8.65 |
| CL589.Contig11_All | calcium-dependent protein kinase 2 | 2.53 | CL589.Contig13_All | calcium-dependent protein kinase 9 | 6.42 |
| CL589.Contig18_All | calcium-dependent protein kinase 2 | -6.56 | CL589.Contig19_All | calcium-dependent protein kinase 2 | 9.54 |
| CL589.Contig3_All | calcium-dependent protein kinase 2 | 5.01 | CL589.Contig7_All | calcium-dependent protein kinase 9 | 1.88 |
| CL8223.Contig12_All | calcium-dependent protein kinase 7 | -2.21 | CL8223.Contig16_All | calcium-dependent protein kinase kinase | 1.08 |
| CL8223.Contig17_All | calcium-dependent protein kinase 8 | -6.05 | CL8223.Contig18_All | calcium-dependent protein kinase 8 | 1.47 |
| CL8223.Contig1_All | calcium-dependent protein kinase 8 | -1.43 | CL8223.Contig23_All | calcium-dependent protein kinase 7 | 7.48 |
| CL8223.Contig5_All | calcium-dependent protein kinase 7 | 1.05 | Unigene12474_All | CDPK-related kinase 4 | -1.08 |
| Unigene13270_All | calcium-dependent protein kinase 3 | 5.76 | Unigene13336_All | CDPKC_ARATH | -5.54 |
| Unigene1527_All | stomatin-like protein 2 | 1.36 | Unigene15948_All | calcium-dependent protein kinase 1-like | -1.99 |
| Unigene16073_All | Monoraphidium neglectum Flagellar radial spoke protein 6 mRNA | -2.07 | Unigene2468_All | calcium-dependent protein kinase 8 | 2.25 |
| Unigene31001_All |  | 1.92 | Unigene31568_All | calcium-dependent protein kinase 20 | -4.75 |
| Unigene34294_All |  | 5.60 | CL2394.Contig1_All | Citrus sinensis UPF0483 protein | -2.09 |
| Unigene6704_All |  | -1.09 | CL3005.Contig1_All | calmodulin-like protein 3 | -3.88 |
| CL21.Contig3_All | dihydrofolate reductase-like | 4.78 | CL3078.Contig5_All | hypothetical protein | 1.19 |
| CL3536.Contig2_All | Calcium-binding EF-hand | 2.54 | CL3536.Contig3_All | calcium-binding protein 49 | -4.43 |
| CL3536.Contig5_All | calcium-binding protein | -2.10 | CL3536.Contig7_All | calcium-binding protein 49 | -3.31 |
| Unigene1061_All | CML9_ARATH | 1.03 | Unigene1159_All | calmodulin-like protein 3 | -1.62 |
| Unigene13512_All | calmodulin-like protein 3 | -1.29 | Unigene13678_All | caltractin-like | 2.60 |
| Unigene17278_All | BCL2-associated athanogene 5 | 1.23 | Unigene19718_All | glycine-rich protein A3 | -1.03 |
| Unigene19959_All | calcium-binding protein 41 | 2.29 | Unigene22195_All | hypothetical protein | -1.07 |
| Unigene23011_All | calcium-binding protein 2 | 1.53 | Unigene286_All | calcium-binding protein 23 | 1.33 |
| Unigene287_All | calcium-binding protein 23 | -3.34 | Unigene3152_All | calcium-binding protein 44 | -1.58 |
| Unigene35995_All | ovarian cancer-associated gene 2 protein homolog | -4.44 | Unigene3781_All | hypothetical protein | 1.78 |
| Unigene5006_All | hypothetical protein | 1.62 | Unigene5187_All | glycine-rich protein A3 | -9.50 |
| Unigene5548_All | resistance protein N-like | 2.30 | Unigene7771_All | polcalcin Phl p 7-like | 1.62 |
| Unigene8148_All | hypothetical protein | 1.53 |  |  |  |
